# Designing a web-application to support home-based care of childhood CKD stages 3-5: Qualitative study of family and professional preferences

**DOI:** 10.1186/1471-2369-15-34

**Published:** 2014-02-18

**Authors:** Veronica M Swallow, Andrew G Hall, Ian Carolan, Sheila Santacroce, Nicholas JA Webb, Trish Smith, Noreen Hanif

**Affiliations:** 1 School of Nursing, Midwifery & Social Work, University of Manchester, University of Manchester, Oxford Road, Manchester M13 9PL, UK; 2 Royal Manchester Children’s Hospital, Central Manchester University Hospitals NHS Foundation Trust, Oxford Road, Manchester M13 9WL, UK; 3 School of Nursing, University of North Carolina at Chapel Hill, School of Nursing 2007, Carrington Hall Campus Box 7460, Chapel Hill, NC 27599-7460, USA

**Keywords:** Interactive health communication applications (IHCA), Self-efficacy, Online parent information and support (OPIS) application, Chronic kidney disease (CKD), Children, Clinical support, Parental care-giving, Long-term

## Abstract

**Background:**

There is a lack of online, evidence-based information and resources to support home-based care of childhood CKD stages 3-5.

**Methods:**

Qualitative interviews were undertaken with parents, patients and professionals to explore their views on content of the proposed online parent information and support (OPIS) web-application. Data were analysed using Framework Analysis, guided by the concept of Self-efficacy.

**Results:**

32 parents, 26 patients and 12 professionals were interviewed. All groups wanted an application that explains, demonstrates, and enables parental clinical care-giving, with condition-specific, continously available, reliable, accessible material and a closed communication system to enable contact between families living with CKD. Professionals advocated a regularly updated application to empower parents to make informed health-care decisions. To address these requirements, key web-application components were defined as: (i) Clinical care-giving support (information on treatment regimens, video-learning tools*,* condition-specific cartoons/puzzles, and a question and answer area) and (ii) Psychosocial support for care-giving (social-networking, case studies, managing stress, and enhancing families’ health-care experiences).

**Conclusions:**

Developing a web-application that meets parents’ information and support needs will maximise its utility, thereby augmenting parents’ self-efficacy for CKD caregiving, and optimising outcomes. Self-efficacy theory provides a schema for how parents’ self-efficacy beliefs about management of their child’s CKD could potentially be promoted by OPIS.

## Background

Chronic kidney disease stages 3-5 (CKD) is a complex set of disorders with a wide range of primary causes and complications [1]. As children and young people (children) will have CKD for life and are at risk of long-term complications, early diagnosis and optimal management are essential [2,3], therefore, skilled, home-based clinical care by parents, supported by professionals is required. Furthermore, parents and professionals often involve older children in their own care in order to promote their ability to take on clinical management responsibility as they move towards independence. Structured materials tailored to parents’ needs are highlighted as key to optimising care of children with long-term conditions, yet childhood CKD-specific, on-line resource provision is variable and unrelaible as it has little evidence-base [2].

A recent Cochrane review of computer-based programmes which combine health information for people with chronic diseases with online peer support, decision support, or help with behaviour change, found they improved users’ knowledge, social support, health behaviours and clinical outcomes, and were more likely than not to improve users’ self-efficacy [4]. There are changing trends in internet use, a dearth of research on what parents think about existing formats of on-line information and support, and a ‘digital divide’ between those who do/do not have internet access [5]. These factors mean that we need a detailed examination of what on-line support individual parents use, would like, and how they and their children think it could best be delivered to meet parents’ variable needs.

In recent research investigating parents’ views of managing childhood CKD, some parents found it challenging to take responsibility for delivering complex aspects of care such as multiple medications via different routes, nasogatric tube feeding, peritoneal dialysis and a restrictive ‘renal diet’. Parents described how they participated in MDT-led, hospital and home based learning and assessment that was both planned (involving allocated clinical lessons and tasks) and spontaneous (in response to current clinical situations) [6]. If parents experienced difficulty processing health information (a concept defined as Health Literacy [7]) this sometimes meant they did not recognise important clinical changes, potentially contributing to impaired kidney function for their child. However, some parents were reluctant to acknowledge comprehension difficulties in case their parenting skills were criticised by professionals [8], thus increasing their likelihood of web-searching for CKD specific information and support. Parents wanted to be empowered to safely and effectively manage clinical responsibilities at home [9], with fathers and mothers often having differing information and support needs [10].

While parents of children with CKD positively evaluated professionals’ face-to-face, telephone and email support, they also identified a need for continuously available, accessible, reliable on-line support to empower them to deal with uncertainties as they arise [8]. Parents also indicated a need to enhance self-efficacy (the extent to which they perceive themselves capable of performing a specific set of tasks [11]) in caring for their child and minimise the need to ‘bother’ renal professionals (for example at night or weekends) for reminders or advice. Some parents purchased computers specifically to search the internet for CKD-specific information, and as children use the internet at an increasingly young age, some being more confident at this than parents, some parents relied on children’s help to seek online CKD-specific information [8]. However, parents and moreover children can have difficulty discerning trustworthy sources of online information from those that are not. Therefore, involving families in creating and evaluating new on-line material that is reliably available and trustworthy should help meet parents’ needs and preferences.

The research reported here is the initial phase of a larger study that is developing and evaluating an online parent information and support (OPIS) application for parents of children with CKD stages 3-5 [12]. Working with patients, parents and the multidisciplinary team (MDT) in one UK children’s kidney unit, the project aims are: Phase 1, to identify desirable components for OPIS; Phase 2, to develop resources that address the identified needs; and Phase 3, to conduct a pilot, feasibility randomised controlled trial of usual professional support versus usual support supplemented by OPIS to inform a future protocol for a full national trial of OPIS.

### Theoretical framework

Because of the need identified in the literature for individual support for parents of children with long term conditions [10,13,14], as well as family-management support [15], and because computer based programmes are more likely than not to improve users’ self-efficacy, we used Bandura’s Self-efficacy theory [11] to help organise and explain our findings in this initial phase of web-application development. The theory proposes that an individual’s belief in their own self-efficacy comes from four main informational sources: mastery experiences (i.e., practice, role play), vicarious experiences (i.e., observing others enacting the behaviours), verbal persuasion (i.e., listening to others explaining the way they successfully manage certain experiences), and physiological (i.e., rapid heart rate) and affective (i.e., anxiety) states. In the context of developing a web-application for parents of children with CKD, self-efficacy theory, therefore, provides a schema for how parents’ self-efficacy beliefs about family self-management of their child’s CKD could potentially be promoted by OPIS [11].

## Methods

### Design

This article describes the analysis of data obtained during qualitative interviews in Phase 1 in which parents, patients and professionals talked about the types and forms of information they would like included in OPIS. These results are being used in Phases 2 and 3 when developing and evaluating OPIS.

### Study setting

The research was undertaken in a children’s kidney unit in the North of England, part of a network of 13 units in the United Kingdom.

### Ethical approval

Approval to conduct the study was obtained from the NHS Research Ethics Committee (Reference: 11/N/W/0268) and the NHS Trust Research and Development Department.

### Recruitment

To achieve an in-depth understanding of participants’ views on the proposed web-application we undertook a qualitative study [16]. To achieve maximum sampling variation, patients with CKD stage 3-5 were identified from unit records using purposive sampling based on age, sex, ethnicity and CKD stage; their parents were invited to participate by the researcher (IC) after confirming they had a home PC and internet access; parents of children aged 0-4 years were also invited but because their children were considered too young to participate, these parents’ provided proxy accounts on behalf of their children. Patients considered old enough to participate were, with parents’ permission if appropriate, invited to take part; and all MDT professionals in the unit and ward were invited to participate.

Recruitment took place between December 2011 and April 2012. Study information was presented to patients according to age and level of understanding. Parents, patients aged over 16 years, and professionals provided signed consent after receiving written and verbal explanations; verbal consent (and where appropriate, written assent) was obtained from patients aged 5-19 years, after parents read information sheets/consent forms with them. Because the study involved only one children’s kidney unit, information about diagnoses and age which could lead to participant identification is omitted from the reporting.

### Data collection

We undertook individual and/or group semi-structured interviews to explore individuals’ views and experiences. Interviews were conducted by IC in the family home or in a quiet space in the hospital. Initially we aimed to conduct only focus groups but found that some interested parent and professional participants were unable to participate because of last minute, competing demands on their time in relation to patients’ clinical needs. Therefore, we offered both interviews and focus groups to accommodate respondents’ changing circumstances; this enabled all those who were interested to participate.

Child-friendly, age appropriate interview techniques such as ‘draw and tell’ were used when appropriate and only to stimulate discussion with young children, therefore, drawings were not analysed. An interpreter was available if needed for participants whose first language was not English but no participants required this service. Interviews were informed by topic guides and were digitally recorded, transcribed verbatim and anonymised. The topic guides were devised by the research team, drawing on clinical and research knowledge and experience. Specific topic guides were developed respectively for parents, professionals and patients aged 5-8 years, 9-12 years and 13-19 years; these included a range of topics and prompts addressing the following broad areas: health information needs, problems with health information, sources of health information, suggested delivery methods and format of health information. Please see Additional file 1 for examples of the respective topic guides for group interviews, these were adapted as appropriate for individual interviews. Discussion within the interviews included prompts to elicit perspectives about issues relating to self-efficacy, in particular the four main informational sources: mastery experiences, vicarious experiences, verbal persuasion, and affective states.

In focus groups, an observer or the researcher took field notes on interactions between the group members and these informed our analysis. For example, it was noted that some parents did not participate in discussion initially but as the discussion proceeded they seemed to be prompted by hearing the way others expressed their views, and subsequently articulated their own views within the group.

### Data analysis

Data were analysed using Framework Analysis, a systematic and rigorous approach to qualitative data analysis [16]. Two researchers (VS and IC) independently read and coded the first transcripts, searching for patterns in the data, mapping connections within and between patient, parent and professional data, and seeking explanations for patterns before comparing and discussing these until a consensus was reached; this resulted in the final framework that was then applied to all transcripts, IC then worked independently with the remaining transcripts. Each coded transcript was analysed and pertinent information was transferred to a Microsoft Excel spread sheet for charting where quotations were labelled for retrieval during reporting.

As data management proceeded, emerging themes supplemented interview topics; this iterative process involved moving backwards and forwards between the Framework stages [16,17]. This helped identify new lines of enquiry to pursue during on-going data collection and analysis. Constant comparison of data within and between themes, supplemented by regular discussion with the research team/steering group opened up meaning in the text until no new themes emerged [16] and theoretical saturation was reached [18] when recruitment ceased. To ensure trustworthiness and credibility, reduce potential bias and enhance theoretical sensitivity we incorporated reflexivity into the data management process regularly considering whether analysis might have been compromised through premature closure in favour of our own preconceived ideas [16,19]. Because the study involved only one site, information about patients’ age or diagnoses that could lead to easy identification is omitted from the reporting and instead of using the term child or young person we use the generic term ‘patient’. Analysis revealed a framework of themes and sub-themes relating to participants’ requirements for the proposed web-application, these form the basis of the following section where quotations are juxtaposed with discussion to illustrate the results from parent, patient and professional perspectives.

## Results

### Sample description

A total of 70 participants (26 patients, 32 parents and 12 professionals), i.e. 37.6% of the 186 (30 patients, 44 Parents and 42 professionals) invited, were recruited to phase 1. Tables 1, 2 and 3 describe participants’ characteristics.

**Table 1:** Variables and additional characteristics of patient participants

**Variable**	**Participants total n = 26**
**Patient age (years)**	**n=**
5-10	7
11-15	10
16+	9
**Gender**	
Female	12
Male	14
**Ethnicity**	
White British	15
South Asian	11
**CKD stage**	
3	9
4	6
5	11

**Table 2:** Variables and additional characteristics of parent participants

**Variable**	**Participants total n = 32**
**Gender**	**n=**
Female	23
Male	9
**Ethnicity**	
White British	25
South Asian	7
**CKD stage of child**	
3	9
4	16
5	7

**Table 3:** Variables and additional characteristics of professional participants

**Variable**	**Participants total n = 12**
**Gender**	**n=**
Female	8
Male	4
**Ethnicity**	
White British	9
South Asian	3
**Occupation**	
Nurse	5
Doctor	3
Dietitian	2
Play specialist	1
Pharmacist	1

Data were collected through: 26 individual patient interviews (15 patients were interviewed with their parents present, there were 14 parents present at these interviews as one family included twin patients); 32 parent-only interviews; three parent focus groups each involving two parents from different families; one focus group with six professionals, and six individual professional interviews (representing dietetics, medicine, nursing, the play service and pharmacy).

Overall, participants’ accounts allude to two key components of the proposed web-application to improve parental self-efficacy for family management of CKD:

(i) Clinical care-giving information

(ii) Psychosocial support for care-giving.

#### Clinical care-giving information

During the interviews, members of all groups identified a lack of reliable, evidence-based, information on the internet to support family management of CKD, and recommended that OPIS contain condition-specific material that is continously available, reliable, accessible and user-friendly. Parents emphasised the importance of being able to access accurate trustworthy information and communication tools that facilitate interaction with individuals in other families living with CKD. Furthermore, professionals were enthusiastic about the scope for providing standardised information for parents to access online, believing this to be a way of enhancing professionals’ communication with parents, whilst helping to relieve pressures on staff. All groups wanted the application to explain about, demonstrate, and enable home-based clinical caring; in addition, professionals wanted material to be regularly updated, and to give parents confidence to make informed health care choices.

Clinical care-giving information requirements for OPIS included information about CKD and treatment regimens, videos containing CKD-specific information, CKD-specific educational cartoons and puzzles, and a question-and-answer area.

### Information on CKD and treatment regimens

In addition to clear information on the range of conditions that can lead to CKD, parents suggested that OPIS should include information on issues such as renal diets, symptom management, medication management and treatment side-effects. In particular parents wanted evidence-based facts on renal diets because:

There is very little information concerning a renal diet and making sure you’re cooking the right foods…it’s also contradictory what you do find (Mother/014).

Professionals’ accounts indicate that they were very aware of the challenge that renal diets can pose for patients and parents; a lack of web-based information and the fact that existing renal cookbooks tend to be more adult-orientated led to suggestions from professionals and families for renal recipes on OPIS and:

…a paediatric cook-book would be great (Professional/119).

Nevertheless, professionals also recognised that it is difficult for some families to adhere to the renal diet regimen, particularly if the parents have no interest in cooking.

Furthermore, professionals, patients and parents suggested that OPIS could include information about the importance of compliance with treatment regimens and the potential risks of non-compliance. There were differences expressed with professionals stating that non-compliance is a considerable problem and parents, children and young people presenting mixed views about this issue:

Patient [non] adherence to taking medication is a huge problem for the medical team (Nurse/103).

I understood what to eat and not to eat. Sometimes I follow [the advice], sometimes I don’t! (Young Person/007).

Patients [are] disinterested and potentially [in] denial that they have the condition (Mother/014).

Patients, meanwhile, highlighted that knowing how to recognise the significance of certain symptoms would be very useful to help them manage the risk of CKD deteriorating. Patients believed that potential side-effects of treatments such as haemodialysis, as well as medication management and side-effects, and invasive procedures that may alter their body’s appearance should be discussed on OPIS:

… the fistula [vascular access for dialysis] is one [procedure] I would like to see [on OPIS]. … it was quite scary to think that it would look like that…it would just be massive in your arm… and possibly how the tube [gastrostomy] would look - the tube that goes into your stomach (Patient/047).

It would be good if they could say have a section with tablets and you can take it in this, this and this form (Patient/047).

### Videos containing CKD-specific information

A recurrent issue across interviews was the potential value of learning-videos presenting CKD-specific information. By demonstrating how to undertake procedures at home, participants thought that videos may provide a safety net (preliminary source of information for families anticipating learning how to do procedures, or a review for parents to refer to after receiving initial in-person instruction from a professional). Typically, the procedures suggested included: managing peritoneal dialysis; preparing and administering erythropoietin or growth-hormone injections; and managing naso-gastric tube feeding, renal diets and medications.

…because people learn in different ways, many prefer visual information to the written word *(*Professional/097).

If there was a video we wouldn’t have to wait until the nurse visited to find out what lay ahead with dialysis (Parent/015).

Professionals regarded continuous access to learning-videos as a way of promoting parental self-efficacy by allowing parents to use information whenever they wished by replaying, pausing and rewinding videos at their own pace, as often as needed:

…videos might give parents confidence to make informed decisions on their child’s health, rather than relying on the renal team solely (Professional/075).

Professionals also thought video-learning could help parents understand medication side effects and the risk of acquiring infections such as peritonitis, as well as helping parents explain treatments to their children:

…could use existing resources such as Mickey the Dialysis doll and the play worker’s knowledge to develop video-learning tools (Professional/114).

Professionals thought learning-videos could be particularly helpful to enable non-English speakers to learn to perform clinical procedures at home. Moreover, professionals thought video-learning would help reduce costly mistakes caused by parents who may be unsure about treatment regimens/procedures, or they may reduce the number of telephone calls from parents to professionals requesting reminders. A father suggested that video-tools explaining medicines management could improve adherence.

### CKD specific, educational cartoons/puzzles

Both parents and patients suggested CKD specific educational cartoons and puzzles to support parents’ efforts to explain CKD to their children. Parents specifically suggested a cartoon about having a transplant to help explain what is actually happening in a patient-friendly way:

…because children learn from them [cartoons] on the television so much (Mother/159).

…[to explain to the patient] why he’s different to other children (Parent/016).

Cartoons are a great way to explain to children the effects of CKD (Father/015).

Older patients also recommended CKD specific, educational cartoon strips as a way of enabling parents to engage young children in learning about CKD. In particular, patients indicated a preference for cartoons that involved kidneys, suggesting OPIS could:

Introduce characters that help to explain the disease e.g. Karl the Kidney (Patient/047).

Other patients suggested CKD specific cartoons based on well-known characters such as Peppa Pig and Monsters Inc. Meanwhile, parents suggested adapting Tom and Jerry cartoons as a way of attracting children to use interactive activities on OPIS.

### Question and answer area

Parents observed that while professionals are not available all the time, many questions arise when parents are at home; therefore, several suggested a question and answer (Q&A) forum including an online ‘post-box’ and an area to facilitate consultation with staff. However, limitations were acknowledged with these suggestions:

An interactive Q&A forum would be useful but how would it work and who would moderate it and answer the questions; how long would they take to reply? (Father/015).

Other parents suggested a frequently asked questions (FAQ) document. Professionals also suggested interactive Q&A forums because parents may worry in the middle of the night, or forget to write down their questions and concerns before attending clinic. This forum could address parental concerns about minor issues before they lead to bigger ones, may reduce anxieties generated by discussion with other parents, and could reduce unnecessary communication with staff, thereby relieving pressure on staff. Patients also recommended a Q&A area to prompt answers from staff that the internet does not provide, although patients recognised that some parents may not want online information:

…a FAQ page on the Q&A Forum with the promise of providing an answer to queries within a week (Professional/083).

…some parents may prefer a phone call or face to face communication (Patient/003).

#### Psychosocial support for care-giving

Psychosocial support requirements for OPIS included social networking, managing stress, enhancing health care experiences, and family case studies.

### Social-networking

A social networking forum was suggested by professionals who thought it would be a natural extension to that which occurs in clinical settings:

So it wouldn’t be a big leap to have online networking, it would help more than hinder (Professional/114).

However, professionals acknowledged the need for a forum to be carefully moderated because:

Some parents who socially network can be irresponsible; administering treatments to their children they have heard of from other parents (Professional/119).

Although Facebook and other online social networking forums exist, several parents said they would prefer one specific to CKD because:

I am a bit worried about being involved in the wrong one [forum] (Parent/035).

Parents highlighted that a CKD specific, social networking forum would enable them to discuss care-giving issues with other parents of children with CKD; parents also thought this would help children feel less isolated by being able to be introduced by their parents to other children with CKD (as their school friends may not understand and children with CKD want to feel ‘normal’), moreover it:

…would be really useful for parents to have a social network with other parents to talk about the stress of managing the condition with people that know (Mother/149).

Polarised views occurred between children and young people in favour of social networking, and mixed views with the professional and parent groups. Some parents and professionals appreciated the immense value of families being able to communicate with a wider circle of like-minded peers. Other parents were concerned about the inherent risk of vulnerable children providing personal information to strangers and the risk of inaccurate exchanging of information between families. The following quotations illustrate these differences:

I think it’s nice as well if you can talk to someone who’s actually been through it (*Patient/124).*

Maybe if they [patient] talked to somebody who understands, and you [can] make friends (*Parent/149).*

I tend to avoid social networks as much as possible. My daughter uses it, but it seems to be used by paedophiles and bullies (*Father/004).*

I’ve come across parents whose child has had condition X which in that individual is going to be very mild who have been scared to death by talking to parents of the child (*Doctor/097).*

Parents will contact each other with or without online social networking (Nurse/109).

### Managing stress

Parents and patients saw the great potential for OPIS to provide information about strategies for managing the stresses associated with living with CKD; these included coping with the ‘highs and low’ of children’s changing conditions, financial and emotional pressures, and fear about what the child’s treatments might entail, as one father vividly explained:

The terrifying thing is when you first start, ‘what is a dialysis machine?’ We all know, because we see it on the telly [television], that whole thing terrified my wife completely. So she said, “We can’t do that’ and she was terrified (Father/127).

It will be really important to describe the process of going through a transplant, the checks and procedures (Patient/003).

Fears about the future were highlighted by patients and parents, and several parents were concerned because their child was unwilling to adhere to the restrictions of the renal diet and the complex and often unpleasant medication regimen. Poor adherence was an increasing concern for parents as children moved into adolescence and began to assert their independence.

Several parents referred to stresses linked to their child’s schooling, for example, because their child’s academic achievements at school are often delayed due to complex medical needs, or because schools do not understand what is involved for patients and parents in managing chronic illness and why CKD children sometimes have poor school attendance. School related stress can also be compounded by pressure from head teachers who want the school to achieve a high standard of academic results and therefore place pressure on parents to ensure children attend regularly and achieve their full potential educationally. Parents however, prioritise their children’s health over their education.

Other stresses included the fact that some children had multiple other health problems as well as CKD. Some parents reported socioeconomic stressors, for example:

Changes to the welfare system are going to place stress on families who have to decide whether it’s worth going to work or becoming a primary carer for their child instead (Father/010).

It would really help to have financial and emotional support advice [on OPIS] (Parent/167).

Finally, a patient reported that stress could be compounded by needing to attend hospital for haemodialysis and would find access to CKD-specific information on OPIS during these times helpful to minimise stress as:

…dialysis is a long and isolating process, it’s a long day [in hospital] (Patient/013).

### Enhancing families’ health-care experiences

Several participants believed that overall, OPIS could significantly enhance families’ experiences of using health services:

Because it would be a single trusted web site (Mother/031).

One mother thought:

It would be great to have online, positive news stories, e.g. charity events and about previous patients who are doing well; children don’t always want to ask their parents or even doctors about problems they might be having, OPIS would help answer those questions (Mother/149).

It would be useful to provide information to parents regarding waiting times and resources they may need to bring when coming into the hospital (Father/004).

An appointment reminder system was recommended because:

Remembering appointments is very difficult; a reminder would be helpful (Patient/013).

Recordings of consultations with professionals would help parents to inform family members of the condition, while also being:

…exceptionally useful to remind yourself of what the doctor said because you often forget (Patient/190).

A professional also suggested producing a video for paediatric patients transferring to adult services as a way of alleviating stress on patients and parents at transition.

The way OPIS is visually presented was discussed although parents and patients were divided between the need for the site to be bright, colourful and interactive, and the importance of accurate and plain information provision.

OPIS should be eye catching and colourful for kids and informative (Patient/007).

The site must be colourful, have lots of sound and interaction (Mother/035).

The look of the site is not as important as the information on it (Patient/038).

Keep the look of the site simple (Mother/159).

Professionals felt that the structure and usability of the site should be established before the look of the site was created:

It’s important to develop something that works first and to create the right impression from the first use of it (Doctor/097).

### Family case-studies

Parents suggested that access to case studies from other families would be useful to provide a parent’s point of view on how to cope, how they manage their child’s condition, to demonstrate that there can be a positive outcome, and so that parents could help their children realise that there is a future after a transplant:

A series of case studies illustrating different children’s lives would be really useful (Father/015).

Parents also thought case studies on OPIS would help to reduce feelings of isolation for patients:

Testimonials [case-studies] are a really useful way for children to understand their condition and realise they’re not on their own (Mother/029).

The potential value of case-studies was also highlighted by patients:

It’d be good to show a patient from the beginning stage right through to transplant (Patient/045).

One patient said they would like to be involved in producing a patient’s diary entry to show a week in the life of a kidney patient.

The supportive benefits of case studies was also highlighted by professionals as a way of helping parents compare their child’s condition with other parents’ accounts:

It would be useful to have case studies or testimonials from adult patients who have transferred from the children’s hospital to give children their story and context (Professional/118).

In summary, our findings have highlighted patients’, parents’ and professionals’ requirements for an online, CKD-specific information and support application for parents, and the way self-efficacy theory can be used to help illuminate the potential value of online resources. The findings illustrate a general consensus in the research cohort on the majority of themes. However there were mixed and polarised views regarding some issues. For example, on the issue of social networking, patients were in favour of them whereas there were mixed views from parents and professionals. Regarding compliance with treatment regimens, professionals and some parents highlighted this as a considerable problem whilst patients appeared to be ambivalent about it. In relation to the visual presentation of OPIS differences between the groupings were less clear, although participants were divided between the site being bright, colourful and interactive, and OPIS providing accurate and plain information. Professionals felt that the structure and usability of the site should be established before the look of the site was created.

## Discussion

Government policies and clinical guidelines acknowledge that parents of children with CKD perform the vast majority of complex and demanding clinical care at home, with remote support from professionals [2,20]. The fact that parents increasingly use the internet to supplement professional support is also widely recognised [21,22]. However, reports highlight the fact that existing online, condition specific, care-giving support and information for parents lacks a rigorous evidence base [23], therefore, some parents are wary of acting on information they find online in case they endanger their child’s health and wellbeing [8].

In this paper, we have developed an account of parent, patient and professional views on the components required in a proposed web-based application to address parents’ information and support needs in CKD management and thus promote parental confidence in their caregiving ability. We used a framework based on self-efficacy, as conceptualised by Bandura, that to our knowledge has not previously been used in the context of childhood CKD. Using the self-efficacy theory has helped to illuminate the way parents’ self-efficacy beliefs can be optimised. Self-efficacy is the extent to which an individual feels able to complete tasks and reach goals and provides a basis for personal motivation, well-being, and feelings of accomplishment. Furthermore, self-efficacy can affect the extent to which individuals experience distressing emotions and experience desirable outcomes [11,24]. In the context of parental caregiving for the child with CKD, self-efficacy can play an important role in helping to determine whether the parent perceives caregiving as a threat or a challenge, and avoids or invests in developing required skills and problem-solving strategies.

**Figure 1:**
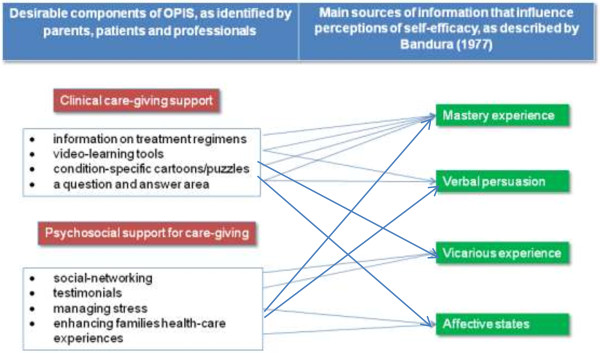
Model of online resources to promote parent self-efficacy for CKD caregiving.

The four main sources of information that influence perceptions of self-efficacy [11]) when integrated with the two main components of the web application identified through our data analysis have resulted in the Model of Online Resources to Promote Parent Self-Efficacy for CKD Caregiving (see Figure 1).

Conceptual models are iterative documents that evolve as the practice issue and/or the context changes. Our model was developed iteratively to provide a clear visual depiction of the intersection of key aspects of the resource needs and preferences identified by participants. The model demonstrates the connections between the factors that can support collaborative development of on line resources, in a manner that makes sense to professionals who may wish to adopt a similar approach. We verified the model by using it with stakeholders to explain the practice issue.

Connections between the main sources of information that influence perceptions of self-efficacy and the desirable components of OPIS as identified by parents, patients and professionals provide a conceptual framework that acts as foundation for the development of OPIS. The components of this model have, therefore, informed the development and current evaluation of OPIS. Future study will aim to refine the model so that it provides a conceptual framework for planning ongoing OPIS development.

*Mastery experience* relates to actual performance of a task and is believed to be the most powerful source of information influencing self-efficacy [11]. Successful performance of a skill or task can promote self-efficacy whereas repeated failures may lower self-efficacy; however, success can be attributed to an individual’s own efforts and abilities. In the context of providing care for the child with CKD, parents, patients and professionals identified information provision on treatment regimens (in particular through video-learning tools, condition-specific cartoons/puzzles, and a Q&A area) as key to parents mastering CKD management. This approach is consistent with Knafl’s recommendation that researchers focus on developing and testing interventions to help parents of children with long-term conditions master treatment regimens and fit them into their everyday life [25] and reinforces the importance of improving services iteratively [26].

According to Bandura [27], *verbal persuasion* is evident in any situation when individual/s attempt to convince others that they have the ability to accomplish a task. In our study, professionals, parents and patients talked about the potential value of a FAQ or Q&A area (both synchronous and asynchronous) and video-learning tools; the value of these approaches was perceived to be two-fold: (i) to promote parents’ understanding about CKD management issues as they arise, and (ii) enable their own belief that they can deal with certain clinical issues based on the information provided in OPIS, or in situations where they have previously been coached in procedures (e.g. injection technique, gastrostomy feeding, peritoneal dialysis) or received clinical explanations from professionals (e.g. renal diet advice, principles of medication management), but have difficulty recalling the exact detail of the instructions. These approaches could help parents determine if their concerns are of a nature that requires immediate and direct contact with a professional, or reduce their need to contact professionals by telephone/email with non-urgent queries.

Moreover, information and verbal encouragement provided in a Q&A area and through video-learning tools providing step-by-step demonstrations of clinical procedures are likely to be viewed positively by parents and patients, and may help increase parents’ self-efficacy for achieving CKD management outcomes.

Observing or talking with others in a similar situation, or *vicarious experience*, can raise or lower self-efficacy expectations depending on the success or failure of the role models concerned [11]. However, to effectively raise self-efficacy, it is important for role models to be as similar to the observer as possible on variables such as age, sex, or condition. Therefore, by developing a CKD specific web-application that addresses the typical concerns, needs and preferences of parents caring for children with CKD, our Model of Online Resources to Promote Parent Self-Efficacy for CKD Caregiving aims to enhance parents’ feelings of self-efficacy, in particular in situations where the individual has no prior experience on which to base judgments of capability. Social networking and access to case studies from families living with CKD and providing clinical care at home were key recommendations from participants. A crucial aspect of learning by vicarious experience is that role models need to be seen as facing and overcoming CKD-related caregiving threats/challenges through determined efforts rather than with ease [28]. Therefore, observing, hearing or reading about the real experience (the *highs* and the *lows*) of other parents managing experiences such as undergoing a kidney transplant or managing home-based renal diets, may serve to increase parents’ self-efficacy.

*Affective states* such as parental anxiety about their child’s future, and concerns about what to do if their child is unwilling to cooperate with unpleasant treatments and restrictive diet and fluid regimens may lower parents’ perceptions of self-efficacy. In the same way that parents in our previous research exploring the way families learned to manage childhood CKD reported feelings of stress, anxiety and physiological arousal when faced with home-based clinical responsibilities, parents in the current study described concerns surrounding their child’s condition, treatments or future wellbeing [6,8]. According to Bandura [11,27,29], perceptions of threat versus challenges in stress-situations can provoke physiological arousal and negative affective states. Experiencing these symptoms can lead to further doubt in their competence at performing tasks and lower self-efficacy. Therefore, by providing a credible, clinical and psychosocial information and support tool, OPIS has the potential to help parents develop positive self-efficacy beliefs.

Our data have demonstrated both similarity to and divergence from the literature concerning family management of long-term conditions. This study’s primary contribution is in applying the concept of self-efficacy to CKD management, thereby potentially assisting parents to safely master CKD caregiving for their child and effectively integrate it into family life. As reported earlier in this paper, a recent Cochrane review of computer-based programmes which combine health information for people with chronic diseases with online peer support, decision support, or help with behaviour change, found that they were more likely than not to improve users’ self-efficacy [4]. The review recommended further high quality studies to determine the best type and way to deliver Interactive Health Communication Applications (IHCA) programmes, and to establish how IHCAs influence different groups of people with chronic illness. This paper describes and discusses phase 1 of a three phased, mixed methods study that is developing and evaluating an IHCA for parents of children with CKD [30]. Our study, therefore, addresses recommendations from the Cochrane Review and contributes to the literature in this emerging field of online health care support.

Our study also addresses a gap in the existing literature through a methodological and conceptual approach that is novel in the field of parental caregiving for a child with CKD. Therefore, we argue that this paper makes a methodological and conceptual contribution to knowledge in this area. Previous studies, including our own, mostly drew on data collected from parents whose knowledge and skills for CKD caregiving were well established, and who had developed their own unique approach to caregiving [6,8,15]. The OPIS will provide resources for parents as their child enters the CKD trajectory, thereby providing continuously accessible, rigorously developed online resources should they at any time wish to supplement the information and support provided by professionals. There is little prior evidence of researchers’ collaboration with families and professionals to develop and evaluate parent information and support resources in the form of an IHCA and/or using the progressively focused approach we adopted in this study. The key theoretical contribution of this paper is its focus on self-efficacy [11]) and the potential for this concept to help us shape, develop and evaluate a complex online parent information and support intervention [30].

A limitation of this study is its focus on parental caregiving for children with only one condition; however our design could potentially be transferrable to the management of other conditions, as is usual in qualitative research. Furthermore, this study was conducted in only one children’s kidney unit. This limitation means that further study is needed in other children’s kidney units to refine the core set of generic resources created in this pilot in order to make them applicable to the wider UK population of children with CKD stages 3-5. In addition, unit specific resources will need to be developed (for example videos showing a nurse teaching peritoneal dialysis management for the home may demonstrate slightly different equipment/techniques in each unit). Moreover, although self-efficacy theory is a valuable conceptual framework it may also be a limitation of our study as Bandura cautions that rather than high self-efficacy following failure or setbacks, individuals might have low self-efficacy that causes them to lose faith in their own capabilities and to develop increased stress and depression [29]. Further studies should, therefore, address this issue. A further limitation is that duration of CKD of patients at time of assent/consent of family members was not collected in this phase of the study. Whilst recruitment to this phase was encouraging given the extensive clinical and personal time demands on the participants invited, we may have reduced the proportion of decliners by offering telephone interviews as an option. These have been demonstrated to be particularly useful for busy clinicians, patients and parents [31-33].

## Conclusion

In summary, through working collaboratively with parents, patients and professionals, and by employing a conceptual framework that explicitly acknowledges the importance of promoting parents’ self-efficacy, we can offer new insights into the online support and information needs of individual parents within families managing home-based care of their child’s CKD. These insights highlight the significance of mastery experience, vicarious experience, verbal persuasion and affective states as sources of information that can influence parents’ perceptions of self-efficacy. By combining these sources of information with the desirable components of OPIS (Clinical care-giving information and Psychosocial support for care-giving) as identified by parents, patients and professionals, and by creating the Model of Online Resources to Promote Parent Self-Efficacy for CKD Caregiving this study has provided a responsive framework with which to develop and evaluate the OPIS web-application.

## Abbreviations

CKD: Chronic-kidney-disease; IHCA: Interactive Health Communication Applications; MDT: Multidisciplinary team; OPIS: Online Parent Information and Support; REC: Research Ethics Committee.

## Competing interests

The authors declare that they have no competing interests.

## Authors’ contributions

VS led study conceptualisation, design, the funding application, data collection, analysis and drafted the manuscript; AH contributed to design, the funding application and the manuscript; IC undertook fieldwork (collected and analysed data and contributed to the manuscript); NW contributed to the funding application and manuscript; TS contributed to design, the funding application, fieldwork and the manuscript and NH provided a parent perspective to the study and contributed to the manuscript. All authors read and approved the final manuscript.

## Pre-publication history

The pre-publication history for this paper can be accessed here:

http://www.biomedcentral.com/1471-2369/15/34/prepub

## Supplementary Material

Additional file 1 Topic guides. Click here for file
