# Supplementary material for: Designing a web-application to support home-based care of childhood CKD stages 3-5: Qualitative study of family and professional preferences
Source: BMC Nephrol. 2014 Feb 18;15:34. doi: 10.1186/1471-2369-15-34 (PMC3933188; doi:10.1186/1471-2369-15-34)
Supplement: Additional file 1 — Topic guides. [file 1471-2369-15-34-S1.doc]

**Additional file 1: Topic guides**

**OPIS Topic Guide: 5-8 Years**

The aim of this group will be to explore the views of child patients regarding new on-line forms of parent/patient information and support; the forms of information they would like more of and how these would best be delivered to patients. Other focus groups will include [children aged 9-12 and young people 13-19] parents and professionals.

Views will be sought on the advantages and disadvantages of existing on-line forms of information and support regarding both specific and generic Chronic Kidney Disease issues. The use and accessibility of supplementary on-line material will be explored in order to collaboratively define the components desirable in an on-line package.

The following topic guide is designed to stimulate debate, gain insights and generate ideas to pursue in greater depth and includes demonstrations of existing on-line material, pod casts or quizzes verified by professionals. Topic guides will be regularly refined to reflect emerging issues.

**Welcome and context-setting**

- The Facilitator will introduce themselves explaining who they are and what their role is.
- They will Introduce OPIS, carefully explaining the overall aim of the study.
- The Facilitator will then explain the ‘ground rules’ for the session:
- They will explain that an audio voice recorder is being used to record the discussion but participants will not be identifiable to anyone other than the researcher and the Principle Investigator.
- …There are no right or wrong answers
- …Confidentiality is important and whatever is said in the room stays in it and is not repeated outside to other people.

• …Everyone will have a chance to speak and be heard.

• …The Facilitator will explain how information will be used/shared.

- …Will carefully explain how findings will be fed back to the research sponsor and participants on the project.
- The facilitator will check that everyone is happy to continue and ask if there are any questions from the group.

**Health information needs – general activity**

*Need lots of coloured pens, pencils and paper.*

- The facilitator asks the group to draw a picture that explains how they feel when they come into hospital for treatment and what their favourite and least favourite bits of treatment are? Drawing for 10 minutes. Facilitator may encourage children and describe issues that give context for analysis later.

*Veale’s ideas of Free drawing*

- The facilitator/play specialist asks each child in turn to explain what they have drawn and why they have drawn it. For 15 minutes.
- The facilitator/play specialist asks the group to perform a role play together as a group, each playing a role in a dialysis session such as patient, nurse, doctor and parents. For 10 minutes.

Role Play – as [Hospital Play Specialist] discussed and Veale and Ritchie mention:

- The facilitator/play specialist asks the children to talk about the role play, one at a time to discuss what they liked or didn’t like about it. 10 minutes.
- In the final 15 minutes the children will be invited to look at 3 or 4 web sites that show, games and activity provision on these sites. They will be asked to comment on whether they like them or not and whether they are useful.

**Feedback**

- Thank the group for their attendance and assure them that there contribution will help to inform the development of OPIS.

**OPIS Topic Guide: 9-12 Years**

The aim of this group will be to explore the views of child patients regarding new on-line forms of parent/patient information and support; the forms of information they would like more of and how these would best be delivered to patients. Other focus groups will include parents teenagers aged 13-19 and professionals.

Views will be sought on the advantages and disadvantages of existing on-line forms of information and support regarding both specific and generic Chronic Kidney Disease issues. The use and accessibility of supplementary on-line material will be explored in order to collaboratively define the components desirable in an on-line package.

The following topic guide is designed to stimulate debate, gain insights and generate ideas to pursue in greater depth and includes demonstrations of existing on-line material, pod casts or quizzes verified by professionals. Topic guides will be regularly refined to reflect emerging issues.

**Welcome and context-setting**

- The Facilitator will introduce themselves explaining who they are and what their role is.
- They will Introduce OPIS, carefully explaining the overall aim of the study.
- The Facilitator will then explain the ‘ground rules’ for the session:
- They will explain that an audio voice recorder is being used to record the discussion but participants will not be identifiable to anyone other than the researcher and the Principle Investigator.
- …There are no right or wrong answers
- …Confidentiality is important and whatever is said in the room stays in it and is not repeated outside to other people.

• …Everyone will have a chance to speak and be heard.

• …The Facilitator will explain how information will be used/shared.

- …Will carefully explain how findings will be fed back to the research sponsor and participants on the project.
- The facilitator will check that everyone is happy to continue and ask if there are any questions from the group.

**Health information needs – general activity**

*Need lots of coloured pens, pencils and paper.*

- The facilitator asks the group to tell draw a picture that explains how they feel when they come into hospital for treatment and what their favourite and least favourite bits of treatment are? Drawing for 10 minutes. Facilitator may encourage children and describe issues that give context for analysis later.

*Veale’s and Ritchie’s ideas of Free drawing*

- The facilitator/play specialist asks each child in turn to explain what they have drawn and why they have drawn it. For 15 minutes.
- The facilitator/play specialist asks the group to perform a role play together as a group, each playing a role in a dialysis session such as patient, nurse, doctor and parents. For 10 minutes.

Role Play – as [Hospital Play Specialist] discussed and Veale and Ritchie mention:

- The facilitator/play specialist asks the children to talk about the role play, one at a time to discuss what they liked or didn’t like about it. 10 minutes.
- In the final 15 minutes the children will be invited to discuss web sites, do they think they are useful (show, games and activity provision on these sites). They will be asked to comment on whether they like them or not and whether they are useful.

**Feedback**

- Thank the group for their attendance and assure them that their contribution will help to inform the development of OPIS.

**Young Person 9-12 Yrs Web Links**

<http://kidshealth.org/kid/htbw/_bfs_USmoviesource.html> movie of how the urinary system works

<http://www.childrenfirst.nhs.uk/kids/index.html> – Site dedicated to children from Great Ormond Street.

**OPIS Topic Guide: 13-19 years**

The aim of this group will be to explore the views of teenage patients regarding new on-line forms of parent/patient information and support; the forms of information they would like more of and how these would best be delivered to patients. Other focus groups will include [children 5-7 and 8-12] parents and professionals.

Views will be sought on the advantages and disadvantages of existing on-line forms of information and support regarding both specific and generic Chronic Kidney Disease issues. The use and accessibility of supplementary on-line material will be explored in order to collaboratively define the components desirable in an on-line package.

The following topic guide is designed to stimulate debate, gain insights and generate ideas to pursue in greater depth and includes demonstrations of existing on-line material, pod casts or quizzes verified by professionals. Topic guides will be regularly refined to reflect emerging issues.

**Welcome and context-setting**

- Facilitator to introduce themselves explaining who they are and what their role is.
- They will Introduce OPIS, what the overall aim of the study is and mention the 3 phases of the study.
- They will introduce the objectives for the focus group session (as mentioned above).
- They will explain that an audio voice recorder is being used to record the discussion but participants will not be identifiable to anyone other than the researcher and the Principle Investigator.
- The Facilitator will then explain the ‘ground rules’ for the session:
- …Confidentiality is important and whatever is said in the room stays in it and is not repeated outside to other people.

• …There are no right or wrong answers.

• …Everyone will have a chance to speak and be heard.

• …Facilitator to explain how information will be used/shared.

- …How findings will be fed back to the research sponsor and participants on the project.
- The facilitator will check that everyone is happy to continue and ask if there are any questions from the group.

**Health information needs – general discussion**

- The facilitator asks the group to tell them what information needs they have in relation to Chronic Kidney Disease? *Bearing in mind different stages of care.*
- The facilitator asks the group to tell them what information sources they use, do they work and could they be improved?

The researcher will discuss web links provided to the group at this stage.

**Condition-specific information needs**

**Introduce task:**

After viewing the web sites and commenting all participants are provided with printed A4 sheets showing a simplified version of the main stages of CKD (please see below), marker pens and post-it notes. Three pieces of flip chart paper are attached to the wall each denoting a stage of CKD from 3 to 5.

• “In your groups, please take 10 minutes to use the post it notes and pens to suggest the sort of information you think parents need at each stage of CKD listed here”.

- Please use the flip chart sheets to record your thoughts.

**Feedback**

- Facilitator to encourage comment/response from the other group members regarding the information presented, mentioning what challenges *and opportunities exist.*
- Thank the group for their attendance and assure them that there contribution will help to inform the development of OPIS. Ask them if it would be acceptable to contact them regarding Phase 2 of the project, to test out the resource.

**Ask the group how they found the process.**

**Yong Person 13-19 Yrs Web Links**

<http://www.nhs.uk/Conditions/Kidney-disease-chronic/Pages/Introduction.aspx> - An NHS resource containing several videos on the stages of CKD and ho to manage it.

- [Kidney Disease an Introduction.](http://www.nhs.uk/Video/Pages/CKDintro.aspx)
- [Kidney Disease the Five Stages?](http://www.nhs.uk/Conditions/Kidney-disease-chronic/Pages/Introduction.aspx)
- [What do our Kidneys do?](http://www.nhs.uk/Video/Pages/CKDwhatkidneysdo.aspx)
- [Kidney Disease Diagnosis](http://www.nhs.uk/Video/Pages/CKDfindandmanage.aspx)
- [How to take care of your kidneys?](http://www.nhs.uk/Video/Pages/CKDlifestyle.aspx)

<http://www.gosh.nhs.uk/gosh_families/information_sheets/chronic_renal_failure/chronic_renal_failure_families.html> - Great Ormond Street Pages with information sheets

[http://www.childrenfirst.nhs.uk](http://www.childrenfirst.nhs.uk/) – Site dedicated to children from Great Ormond Street.

<http://www.childrenfirst.nhs.uk/teens/health/conditions/k/kidney_failure.html> Kidney specific for teens and podcast.

<http://www.kidney.org.uk/young@NKF/poster.jpg> _ Young @ National Kidney Foundation Facebook example.

<http://kidshealth.org/teen/your_body/body_basics/kidneys.html?tracking=T_RelatedArticle> - Easy to read diagram for teens.

[http://kidshealth.org/teen/diseases_conditions/urinary/kidney.html#cat20170](http://kidshealth.org/teen/diseases_conditions/urinary/kidney.html#cat20170)

- Podcast and information regarding CKD for teens.

[http://www.dialysisdiaries.com](http://www.dialysisdiaries.com/) – Mentions various patients experience in dealing with Chronic Kidney Disease.

<http://www.kidney.org/kidneyDisease/kidneyQuiz_v2.cfm> - National Kidney Foundation Take your Kidney IQ test. Great for teenagers.

[http://kidshealth.org/PageManager.jsp?lic=1&article_set=54382&cat_id=20607#cat20579](http://kidshealth.org/PageManager.jsp?lic=1&article_set=54382&cat_id=20607#cat20579) – How the body works pictures and words

[http://kidshealth.org/PageManager.jsp?lic=1&article_set=58227&cat_id=20607#cat20579](http://kidshealth.org/PageManager.jsp?lic=1&article_set=58227&cat_id=20607#cat20579) – CKD/Urinary Word Finder

**OPIS Topic Guide: Parent**

The aim of this group will be to explore the views of parent care-givers on: existing and new on-line forms of parent information and support; the forms of information and support parents (and patients) would like more of and how these would best be delivered to parents. Other focus groups will include patients [children 5-10, 11-15 and young people 16+ and professionals.

Views will be sought on the advantages and disadvantages of existing on-line forms of information and support regarding both specific and generic Chronic Kidney Disease issues. The use and accessibility of supplementary on-line material will be explored in order to collaboratively define the components desirable in an on-line package.

The following topic guide is designed to stimulate debate, gain insights and generate ideas to pursue in greater depth and includes demonstrations of existing on-line material, pod casts or quizzes verified by professionals. Topic guides will be regularly refined to reflect emerging issues.

**Welcome and context-setting**

- Facilitator to introduce themselves explaining who they are and what their role is.
- They will Introduce OPIS, what the overall aim of the study is and mention the 3 phases of the study.
- They will introduce the objectives for the focus group session (as mentioned above).
- They will explain that an audio voice recorder is being used to record the discussion but participants will not be identifiable to anyone other than the researcher and the Principle Investigator.
- The Facilitator will then explain the ‘ground rules’ for the session:
- …Confidentiality is important and whatever is said in the room stays in it and is not repeated outside to other people.

• …There are no right or wrong answers.

• …Everyone will have a chance to speak and be heard.

• …Facilitator to explain how information will be used/shared.

- …How findings will be fed back to the research sponsor and participants on the project.
- The facilitator will check that everyone is happy to continue and ask if there are any questions from the group.

**Health information needs – general discussion**

- The facilitator invites each participant to introduce themselves, whether English is their first language, their ethnicity, their name and their experience of Chronic Kidney Disease. Respecting each participants rights to confidentiality & anonymity.
- The facilitator asks the group to tell them what information needs they have in relation to Chronic Kidney Disease? *Bearing in mind different stages of care.*
- The facilitator asks the group to tell them what information sources they use, do they work and could they be improved?

A list of links provided by the researcher are discussed here. The facilitator could ask what links the group use themselves.

**Feedback**

- Facilitator to encourage comment/response from the other group members regarding the information presented, mentioning what challenges *and opportunities exist.*
- Thank the group for their attendance and assure them that their contribution will help to inform the development of OPIS. Ask them if it would be acceptable to contact them regarding Phase 2 of the project, to test out the resource.

**Ask how they found the process.**

**Parent Web Links**

<http://www.nhs.uk/Conditions/Kidney-disease-chronic/Pages/Introduction.aspx> - An NHS resource containing several videos on the stages of CKD and ho to manage it.

- [Kidney Disease an Introduction.](http://www.nhs.uk/Video/Pages/CKDintro.aspx)
- [Kidney Disease the Five Stages?](http://www.nhs.uk/Conditions/Kidney-disease-chronic/Pages/Introduction.aspx)
- [What do our Kidneys do?](http://www.nhs.uk/Video/Pages/CKDwhatkidneysdo.aspx)
- [Kidney Disease Diagnosis](http://www.nhs.uk/Video/Pages/CKDfindandmanage.aspx)
- [How to take care of your kidneys?](http://www.nhs.uk/Video/Pages/CKDlifestyle.aspx)

<http://www.gosh.nhs.uk/gosh_families/information_sheets/chronic_renal_failure/chronic_renal_failure_families.html> - Great Ormond Street Pages with information sheets

[http://www.childrenfirst.nhs.uk](http://www.childrenfirst.nhs.uk/) – Site dedicated to children

<http://www.childrenfirst.nhs.uk/teens/health/conditions/k/kidney_failure.html> Kidney specific for teens and podcast

<http://www.childrenfirst.nhs.uk/kids/health/illnesses/k/kidney_failure.html> 7-11 year olds

<http://www.childrenfirst.nhs.uk/juniors/hospital/index.html> 4-7 year olds general health and games

<http://www.britishkidney-pa.co.uk/forums.html> - An example of an online Forum

<http://www.kidney.org.uk/young@NKF/poster.jpg> _ Young @ National Kidney Foundation Facebook example.

<http://www.rsnhope.org/programs/kidney_talk3.php> - Renal Support Network example of Podcasts available.

<http://kidshealth.org/parent/medical/kidney/kidney_diseases_childhood.html>

<http://kidshealth.org/kid/htbw/_bfs_USmoviesource.html> – The urinary

<http://kidshealth.org/kid/feel_better/things/dialysis.html>

- Interactive Diagram, Children’s Video, web cast and written information. Not dedicated to renal care but good general site. Articles, quizzes and word jumble. “How the Body Works”.

[http://www.dialysisdiaries.com](http://www.dialysisdiaries.com/) – Mentions various patients experience in dealing with Chronic Kidney Disease.

<http://www.kidney.org/kidneyDisease/kidneyQuiz_v2.cfm> - National Kidney Foundation Take your Kidney IQ test. Great for teenagers.

<http://www.kidneyresearchuk.org/__assets/asset726.pdf> - Kidney research UK Cook book.

**OPIS Topic Guide: Professionals**

The aim of this group will be to explore the views of professionals on: existing and new on-line forms of parent information and support; the forms of information and support parents (and patients) would like more of and how these would best be delivered to parents. Other focus groups will include parents, patients [children 5-7, 8-12 and young people 13-19].

Views will be sought on the advantages and disadvantages of existing on-line forms of information and support regarding both specific and generic Chronic Kidney Disease issues. The use and accessibility of supplementary on-line material will be explored in order to collaboratively define the components desirable in an on-line package.

The following topic guide is designed to stimulate debate, gain insights and generate ideas to pursue in greater depth and includes demonstrations of existing on-line material, pod casts or quizzes verified by professionals. Topic guides will be regularly refined to reflect emerging issues.

**Welcome and context-setting**

- Facilitator to introduce themselves explaining who they are and what their role is.
- They will Introduce OPIS, what the overall aim of the study is and mention the 3 phases of the study.
- They will introduce the objectives for the focus group session (as mentioned above).
- The Facilitator will then explain the ‘ground rules’ for the session:

• …There are no right or wrong answers

• …Everyone will have a chance to speak and be heard.

• …Facilitator to explain how information will be used/shared.

- …How findings will be fed back to the research sponsor and participants on the project.
- The facilitator will check that everyone is happy to continue and ask if there are any questions from the group.

**Health information needs – general discussion**

- The facilitator invites each participant to introduce themselves, their name and their specialty role regarding Chronic Kidney Disease. Respecting each participants rights to confidentiality & anonymity.
- The facilitator asks the group to tell them what information needs they believe parents have in relation to Chronic Kidney Disease? *Bearing in mind different stages of care.*
- The facilitator asks the group to tell them what information sources they use, do they work and could they be improved?

A list of links provided by the researcher are discussed here.

**Feedback**

- Facilitator to encourage comment/response from the other group members regarding the information presented, mentioning what challenges *and opportunities exist.*
- Thank the group for their attendance and assure them that there contribution will help to inform the development of OPIS.

**Ask the group how they found the process.**

**Professionals Web Links**

<http://www.nhs.uk/Conditions/Kidney-disease-chronic/Pages/Introduction.aspx> - An NHS resource containing several videos on the stages of CKD and ho to manage it.

- [Kidney Disease an Introduction.](http://www.nhs.uk/Video/Pages/CKDintro.aspx)
- [Kidney Disease the Five Stages?](http://www.nhs.uk/Conditions/Kidney-disease-chronic/Pages/Introduction.aspx)
- [What do our Kidneys do?](http://www.nhs.uk/Video/Pages/CKDwhatkidneysdo.aspx)
- [Kidney Disease Diagnosis](http://www.nhs.uk/Video/Pages/CKDfindandmanage.aspx)
- [How to take care of your kidneys?](http://www.nhs.uk/Video/Pages/CKDlifestyle.aspx)

<http://www.gosh.nhs.uk/gosh_families/information_sheets/chronic_renal_failure/chronic_renal_failure_families.html> - Great Ormond Street Pages with information sheets

[http://www.childrenfirst.nhs.uk](http://www.childrenfirst.nhs.uk/) – Site dedicated to children

<http://www.childrenfirst.nhs.uk/teens/health/conditions/k/kidney_failure.html> Kidney specific for teens and podcast

<http://www.childrenfirst.nhs.uk/kids/health/illnesses/k/kidney_failure.html> 7-11 year olds

<http://www.childrenfirst.nhs.uk/juniors/hospital/index.html> 4-7 year olds general health and games

<http://www.britishkidney-pa.co.uk/forums.html> - An example of an online Forum

<http://www.kidney.org.uk/young@NKF/poster.jpg> _ Young @ National Kidney Foundation Facebook example.

<http://www.rsnhope.org/programs/kidney_talk3.php> - Renal Support Network example of Podcasts available.

<http://kidshealth.org/parent/medical/kidney/kidney_diseases_childhood.html>

<http://kidshealth.org/kid/htbw/_bfs_USmoviesource.html> – The urinary

<http://kidshealth.org/kid/feel_better/things/dialysis.html>

- Interactive Diagram, Children’s Video, web cast and written information. Not dedicated to renal care but good general site. Articles, quizzes and word jumble. “How the Body Works”.

[http://www.dialysisdiaries.com](http://www.dialysisdiaries.com/) – Mentions various patients experience in dealing with Chronic Kidney Disease.

<http://www.kidney.org/kidneyDisease/kidneyQuiz_v2.cfm> - National Kidney Foundation Take your Kidney IQ test. Great for teenagers.

<http://www.kidneyresearchuk.org/__assets/asset726.pdf> - Kidney research UK Cook book.

1. Veale, A.*, Creative Methodologies in Participatory Research with Childr*en, i*n Researching Children's Experien*ce, S. Greene and D.H. . Editors. 2005, Sage: London.
